# Lipid transport across the mycobacterial cell envelope

**DOI:** 10.1107/S2052252521008885

**Published:** 2021-09-01

**Authors:** Ghader Bashiri

**Affiliations:** a Laboratory of Molecular and Microbial Biochemistry, School of Biological Sciences, The University of Auckland, Auckland 1010, New Zealand

**Keywords:** lipid transport, *Mycobacterium tuberculosis*, mammalian-cell-entry proteins, substrate-binding proteins

## Abstract

The mammalian-cell-entry (Mce) proteins of *Mycobacterium tuberculosis* enable the bacterium to acquire lipids from the host cells. Asthana *et al.* [*IUCrJ* (2021). **8**, 757–774] present the first structural insights into the potential assembly of Mce1 and Mce4, advancing our understanding of lipid transport by the human pathogen that causes tuberculosis.

Tuberculosis is a devastating disease that has afflicted humans since antiquity. Known as Phthisis (Greek), the ‘white plague’ or consumption, tuberculosis appears as a common theme in art, music and literature, and has shaped many elements of human social history (Daniel, 2006 ▸). Even today, one-quarter of the world’s population is estimated to carry latent infections by *Mycobacterium tuberculosis*, the bacterium that causes tuberculosis (Getahun *et al.*, 2015 ▸). So why is this disease so recalcitrant to treatment? This is, in part, due to the distinctive cell envelope of *M. tuberculosis*, which provides a physical barrier against antibiotics (Batt *et al.*, 2020 ▸). Furthermore, this envelope also helps *M. tuberculosis* survive attacks by the host immune system (Batt *et al.*, 2020 ▸), allowing the bacterium to persist in a non-replicating (‘dormant’) state in the host cells (Gengenbacher & Kaufmann, 2012 ▸). A key feature of *M. tuberculosis* is its ability to acquire and metabolize lipids, notably cholesterol and fatty acids, from its human host (Wilburn *et al.*, 2018 ▸). These lipids provide the bacterium with the essential carbon and energy sources to maintain viability over many years (Warner, 2014 ▸). Understanding how these lipids are transported into *M. tuberculosis* may expose vulnerabilities that could then be exploited to develop new therapeutic agents against tuberculosis.

Despite the significance of lipid metabolism in the survival and pathogenesis of *M. tuberculosis*, it is not clear how lipids are transported into the cell, with no structural or mechanistic details on mycobacterial lipid transporters being available. The genome sequence of *M. tuberculosis*, and subsequent studies, have identified four homologous mammalian-cell-entry (Mce) multiprotein complexes that are proposed to play crucial roles in trans­locating various lipid molecules across the cell envelope (Cole *et al.*, 1998 ▸; Casali & Riley, 2007 ▸). These membrane-bound assemblies, however, have so far defied structural analysis. In this issue of **IUCrJ**, Asthana *et al.* (2021 ▸) now provide the first insights into the structure and potential assembly of the Mce1 and Mce4 proteins in *M. tuberculosis*.

Mce proteins play crucial roles in *M. tuberculosis* pathogenesis through reimporting fatty acid and mycolic acid (Mce1), and importing cholesterol from the host cells (Mce4) (Pandey & Sassetti, 2008 ▸; Nazarova *et al.*, 2017 ▸). Each *mce* operon encodes proteins with various roles in the formation of their respective Mce complexes, including six Mce proteins (MceA, MceB, MceC, MceD, MceE and MceF) that act as substrate-binding proteins (SBPs) (Casali & Riley, 2007 ▸). The homologous SBPs from *E. coli* (Ekiert *et al.*, 2017 ▸; Isom *et al.*, 2020 ▸; Liu *et al.*, 2020 ▸; Coudray *et al.*, 2020 ▸) and *Acinetobacter baumannii* (Kamischke *et al.*, 2019 ▸; Mann *et al.*, 2020 ▸) have been shown to form hexameric structures, leading to their central role in lipid transport through either a tunnel- or ferry-based mechanism.

The results presented by Asthana *et al.* (2021 ▸) reveal several advances in our understanding of the Mce proteins in *M. tuberculosis*. They used sequence analysis and secondary-structure prediction to show that all SBPs of Mce1–4 display a conserved four-domain architecture. This arrangement comprises an N-terminal transmembrane (TM) domain, the MCE domain, a helical domain and a tail domain of variable size. They also showed that all these individual domains, except for the MCE domain, require detergents for solubility and stability. Interestingly, the full-length and individual domains of *M. tuberculosis* Mce1A and Mce4A are predominantly present as monomers in solution. This was further confirmed by the crystal structure of the single MCE domain present in Mce4A (Mce4A_39–140_), indicating that this domain could not form homo-hexamers due to steric clashes between monomers. This is a notable difference from the previously reported hexameric SBPs observed in *E. coli* (Ekiert *et al.*, 2017 ▸; Isom *et al.*, 2020 ▸; Liu *et al.*, 2020 ▸; Coudray *et al.*, 2020 ▸) and *A. baumannii* (Kamischke *et al.*, 2019 ▸; Mann *et al.*, 2020 ▸). Finally, using small-angle X-ray scattering (SAXS) experiments and structure-based modelling, they showed that the helical domains of Mce1A and Mce4A interact with the detergent micelles, implying that they either interact with the membrane or the lipid substrates.

These results have consequently led to a proposed model on the likely assembly of the Mce proteins in *M. tuberculosis* (Asthana *et al.*, 2021 ▸). Based on this model (Fig. 1 ▸), the six MCE domains of MceA–F SBPs may interact with each other to form hetero-hexamers, with the helical domains of each polypeptide coming together to form a long and hydro­phobic channel for lipid transport. This structure would be held in between the plasma membrane and the cell surface via interactions with the TM domains and the tail domains, respectively. This model resembles the tunnel-based mechanism described in *Ec*-Pqi (Ekiert *et al.*, 2017 ▸), providing the first experimental model towards the Mce-mediated lipid transport in *M. tuberculosis*.

The proposed model by Asthana *et al.* (2021 ▸) establishes a unique foundation for future studies of the Mce multiprotein complexes, elucidating structural and mechanistic details of lipid transport in *M. tuberculosis*. Such endeavours may also facilitate the development of specific compounds to target cholesterol import as a therapeutic intervention, particularly restricting *M. tuberculosis* growth and survival during persistence.

## Figures and Tables

**Figure 1:**
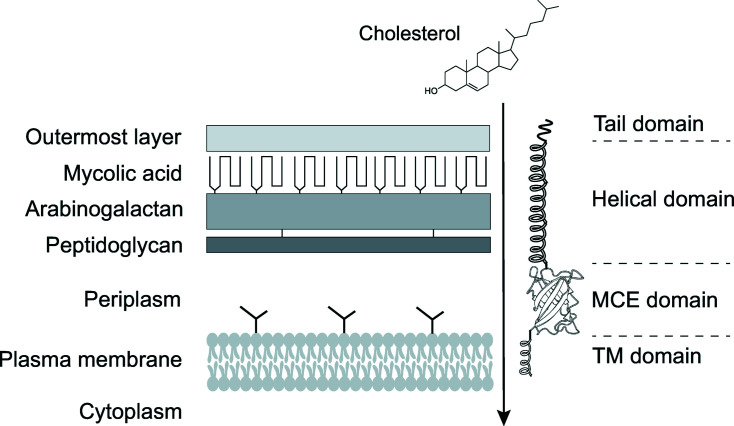
Schematic representation of the results reported by Asthana *et al.* (2021 ▸) within the context of cholesterol import through the *M. tuberculosis* cell envelope. Mce4A is shown to be monomeric in solution, likely forming a hetero-hexameric arrangement with other Mce4 proteins to form a tunnel for lipid transport. A schematic model of the *M. tuberculosis* cell envelope is also shown for comparison, adapted from Chiaradia *et al.* (2017 ▸). The schematics are not drawn to scale.
